# Experiences of the older spousal caregivers of patients with cancer during palliative chemotherapy: a qualitative descriptive study

**DOI:** 10.1186/s12904-023-01313-2

**Published:** 2023-11-23

**Authors:** Kengo Hirayama, Tomoki Kuribara, Miho Oshikiri

**Affiliations:** 1 School of Nursing, Sapporo City University, Kita 11, Nishi 13, Chuo-ku, Sapporo, Japan; 2 Department of Nursing, Sapporo Sato Hospital, 4-10-15, Fushiko 2, Higashi-ku, Sapporo, Japan

**Keywords:** Older spouse, Caregiver, cancer, Palliative chemotherapy, Qualitative study

## Abstract

**Background:**

Several studies have characterized the experiences of family members caring for patients undergoing chemotherapy; however, information about the experiences of older spousal caregivers with intensive caregiving burdens is unclear. Recently, more older patients have been diagnosed with cancer due to the aging population worldwide. Therefore, this study evaluated the patterns in the experiences of older spousal caregivers of patients undergoing palliative chemotherapy for advanced cancer.

**Methods:**

Qualitative research using semi-structured interviews was used in this study involving 10 older spousal caregivers of patients undergoing palliative chemotherapy at a hospital providing advanced cancer care in Japan. The data obtained were analyzed qualitatively and inductively using thematic analysis by Braun and Clarke.

**Results:**

Four themes were identified from the narratives of the participants in this study. The first theme was “getting used to living with the disease,” indicating that the older spouses gradually became accustomed to living with the patient through continued caregiving. The second theme was “deepening view of life and death,” indicating that the older spouses’ views of life and death were deepened by being confronted with patients’ quality of life until death. The third theme was “anxious about the future,” indicating the fear regarding the patient’s progressive diseases and anxiety pertaining to continuing care for the patient while dealing with their health problems. The final theme was “desire for a better rest of life,” indicating that the couple felt their bond was strengthened through caregiving and wishes to live well for the rest of their lives.

**Conclusions:**

The patterns in the experience of older spousal caregivers caring for patients undergoing palliative chemotherapy indicated an aspect of rebuilding their lives as they became accustomed to caregiving, while strengthening their marital bond. The caregiving process involved a mix of emotions, including anxiety about the spousal caregiver’s health problems worsening. However, the caregivers recognized the value of their remaining time. Therefore, they had deep concern for the patient’s comfort, concealing their feelings so that the patient would feel comfortable. This study can contribute to understanding the challenges faced and support needed by older spousal caregivers.

**Supplementary Information:**

The online version contains supplementary material available at 10.1186/s12904-023-01313-2.

## Background

The number of patients with cancer worldwide is increasing annually, with an estimated 23.6 million new cancer cases in 2019 [1]. In recent years, patients with cancer have been living longer because of advances in diagnosis and treatment [2]. However, cancer is Japan’s leading cause of death, and more than 80% of cancer deaths are among older people [3], making this is a global health problem. Furthermore, previous studies have reported that chronic diseases, including cancer, reduce patients’ and their caregivers’ quality of life and function [4, 5].

The importance of family support has been characterized extensively for patients with cancer from diagnosis to the end of life [6]. Previous studies have reported that family caregivers suffer from various issues including poor physical health [7], psychological distress due to anxiety, depression, caregiving burdens [8, 9], and strained social roles [10]. Furthermore, Üzar-Özçeti [11] suggests that family caregivers of cancer survivors with poor psychological skills and abilities were associated with significantly increased caregiver burden and poor quality of life for the caregivers. Moreover, several studies on family caregivers of patients undergoing cancer chemotherapy have reported that caregivers experience psychological distress during caregiving, including grief and stress. This distress can cause physical effects including insomnia and fatigue [12]. In particular, caregiver fatigue becomes more substantial after the patient undergoes chemotherapy [13]. In contrast, over the past few years, many studies have reported that aspects of caregiving were viewed positively as life-enriching experiences for family caregivers, resulting in a more intimate relationship with the patient, personal satisfaction, and increased self-efficacy [14]. Thus, this outlook focusing on advantages of caregiving can lead to psychological adjustment [15]. In consequence, positive aspects of caregiving for patients undergoing cancer chemotherapy have been reported. However, most of these studies were not focused on older adults. Older family caregivers of patients undergoing palliative chemotherapy for advanced and difficult to cure cancer may experience more stress owing to the symptoms associated with cancer treatment and the uncertainty of disease progression.

Currently, the number of older patients with cancer is increasing due to aging populations and extended life expectancies worldwide [16]. Thus, the number of family caregivers supporting cancer patients, especially older spouses, is expected to increase. Senior family caregivers commonly suffer from hypertension, arthritis, and cancer [17, 18]. In addition, caregivers supporting patients with cancer experience the same or higher level of stress compared to that of the patients [19]. Moreover, older caregivers may have more difficulties understanding the patient’s medical conditions and helping the patients due to physical fatigue caused by caregiving, than younger family caregivers [20]. In these situations, they must take on the role of assisting patients with their health problems. In Japan, spouses are the most common principal caregivers who live with the person requiring long-term care [21].

Furthermore, globally, it has been reported that the factors that intensify the burden on older patients’ caregivers are their advanced age and spouses [22]. Research focusing on spouses is an urgent issue for these reasons. Older spouses supporting patients undergoing cancer chemotherapy may face significant burden for providing a convalescent environment, including dealing with adverse events at home. Moreover, the older population may have many negative factors to manage in their living environment. These factors include communication with the patient, psychological distress with caregiving, and the financial burden required for medical treatment [23].

Thus, an in-depth understanding of older spousal caregivers’ experiences and perspective is important to provide suggestions for developing medical care for the older patients with cancer and their families. Therefore, in this study, we evaluated the patterns in the experiences of older spousal caregivers of patients undergoing cancer chemotherapy.

## Methods

### Design

A qualitative descriptive method, including thematic analysis with Braun and Clarke [24], was used based on semi-structured individual interviews. Thematic analysis is the methodology for developing, analyzing, and interpreting patterns across a qualitative dataset [25]. Thus, this method was deemed suitable for this study. This study report followed the Consolidated Criteria for Reporting Qualitative Research (COREQ) guidelines [26] and the tools for evaluating thematic analysis manuscripts for publication [27].

### Patient recruitment and characteristics

Older spousal caregivers of patients with cancer undergoing palliative chemotherapy were included in this study. The inclusion criteria were as follows: 1) ≥ 65 years of age; 2) normal cognitive function; and 3) living with the patients as partners and caregivers and not as parent-child relationships. This is because in Japan, spouses generally represent “married couples” or “couples living together.” The exact definition is used in national surveys conducted by the Japanese government [21]. No restrictions were set for participants regarding chemotherapy drugs or other treatment modalities. Purposive sampling was used to recruit the participants. First, the researcher explained the purpose of the study and the interview eligibility criteria to the physicians and nurses of the cooperating hospitals. Then, they referred the suitable caregivers to the researcher as potential participants. After the researcher informed them of the purpose of the study, the potential participants provided their consent to participate in this study. Finally, the participants who met these eligibility criteria and provided their consent to participate were included in the study. We aimed to select approximately 10 participants for the interviews.

### Setting

Oji general hospital and Tonan hospital were selected as cooperating hospitals. These hospitals provide specialized treatment for patients within the surrounding rural area in Hokkaido, Japan.

### Data collection

The researchers reviewed the questions and developed an interview guide to address the study objectives. Then a pre-interview was conducted using the completed interview guide to verify its validity. Additionally, the interviews were conducted with two researchers, one specializing in qualitative research (KH) and the other trained by the researcher (MO). The main questions in the interview were focused on the caregiver’s daily experiences including questions on his/her feelings at the time and what he/she feels daily. Data on participant characteristics including age, gender, relationship to patient, length of caregiving period, and chronic health problems were also collected during the interviews. Additional file 1 shows the interview guide.

Data collection was conducted from July to December 2019. Face-to-face interviews were conducted in private rooms at each hospital to protect the privacy of the participants. The duration of these interviews were not more than one hour per person. The interview was recorded using an integrated circuit (IC) recorder with the participants’ consent.

### Data analysis

Data analysis was conducted by two researchers with specialization in qualitative research (KH) and some qualitative research experience (TK). The six-phase process of the thematic analysis method was used as a reference for this analysis [24]. First, the interviews were recorded on an IC recorder and converted into written and verbatim transcripts. Each researcher then read the prepared verbatim transcripts multiple times and summarizing them. Next, each researcher reviewed the verbatim transcripts and extracted the passages that addressed the research questions. The extracted content was then abstracted without the loss of semantic content and represented using code. During this process, one semantic content was expressed in one code. Each researcher was trained in coding for uniformity, and then the task of coding was divided among them. Following this, the content was integrated. Thereafter, the codes were read thoroughly and compared to the data, generating categorization based on themes similar to the initial ones. The initial themes of the data and codes were repeatedly examined to ensure consistency. The resulting sub-themes and themes were then iteratively examined and refined until they were finalized and named. As an example of the data analysis process, the development of Theme 1 is represented in Additional file 2.

The continuous variables as age, length of caregiver period and interview time were represented as means using Stata 17.0 (Stata Corp, TX, USA).

The research was conducted with the approval of the Ethics Committee of Hokkaido University (Approval No.19 − 4) and the Clinical Ethics Committee of the cooperating hospitals (Oji general hospital: OGH2019-14, Tonan hospital: 405).

## Results

### Participants’ demographic characteristics

Researchers were introduced to 10 potential participants. After explaining the study to all of them, their consent to participate in this study was obtained. The 10 participants’ demographic characteristics are presented in Table 1. The participants included six women (60%); age of the participants ranged from 65 to 79 years (mean = 70.4). Two participants (20%) were employed, and both were self-employed. All the participants suffered from some chronic diseases, and three of them were cancer survivors. The main consultant and supporters of the participants were family members who lived separately. The interview time ranged from 18 to 60 min (mean = 38.8), and length of caregiving period was ranged from 7 to 48 months (mean = 21.4).

### Themes

From the data, we obtained 152 codes and identified four main themes and 13 sub-themes for each experience of the older spousal caregivers of patients with cancer undergoing palliative chemotherapy. All themes are presented in Table 2.

**Table 1:** Participants’ demographic characteristics

	Age (Y)	Sex	Relationship to patient	Length of caregiving period	Chronic health problems	Patient age (Y)	Interview time (M)
A	60s	Male	Husband	22 months	Lung cancer Oral cancer	60s	18
B	70s	Female	Wife	7 months	Low back pain Bladder cancer	60s	27
C	70s	Female	Wife	7 months	Low back pain Gonalgia	70s	30
D	60s	Female	Wife	32 months	Cataract	60s	35
E	70s	Female	Wife	25 months	Hyperlipidemia Uterine cancer	70s	60
F	70s	Female	Wife	48 months	Low back pain Uterine cancer	70s	47
G	60s	Female	Wife	9 months	Hyperlipidemia Gonalgia	70s	45
H	70s	Male	Husband	13 months	Hyperlipidemia Cataract	70s	44
I	70s	Male	Husband	36 months	Hypertension Prostatic hypertrophy	60s	52
J	60s	Male	Husband	15 months	Arrhythmia	60s	30

### Theme 1: getting used to living with the disease

This theme focused on participants gradually becoming accustomed to living with a patient with cancer by trying to understand the side effects and symptoms associated with the patient’s chemotherapy, trying to reduce the symptoms, and trying to gain medical knowledge for supporting the patient.

#### Habituation of supporting recuperation

Many participants ensured that cancer patients undergoing palliative chemotherapy could recuperate comfortably. This included researching the side effects experienced by palliative chemotherapy patients and then actively preparing meals and doing household chores based on their physical condition and symptoms to reduce their burdens.

*D: “My husband had a tough time at times with little appetite due to the side effects of the chemotherapy. So, I would write down what he ate for breakfast, lunch, and dinner, how much he ate, and things like that.”*

*H: “…used to go shopping with my wife, but now I sometimes go shopping alone. These responsibilities may have increased. I may have become more involved in these areas without realizing it.”*

*C: “At the beginning, when the treatment started, I was the one who devised his diet. However, he didn’t eat much because of the side effects, so we eventually reverted to our previous lifestyle.”*

They also took care of the psychological aspect by respecting the patients’ feelings and tolerating their selfishness so they could be at peace. Furthermore, they tried to hide their feelings.

*G: “I know he is sick, so I try not to get angry and react as gently as possible…”*.

**Table 2:** Emergent themes and sub-themes

Themes	Sub-themes
Getting used to living with the disease	Habituation of supporting recuperation
	Recognition of side effects
	Acquisition of medical knowledge
Deepening view of life and death	Consciousness of death
	Understanding the patient’s experience
	Changing values for treatment
Anxious about the future	Conflicts between treatment efficacy and disease progression
	Mixed feelings of desire to support recuperation and anxiety
	Worry about their own health
Desire for a better rest of life	Values the remaining time
	Energy source for living
	Deepening affection
	Coexistence with society

#### Recognition of side effects

Because of the various side effects symptoms that patients experience with palliative chemotherapy, participants sought to understand these symptoms. They also recognized that symptoms accumulate throughout treatment and can be ameliorated by reducing the dosage of therapeutic drugs.

*B:“Symptoms became progressively more severe in the fourth therapy session than in the first.”*

*I: “When taking the maximum dose of Giotrif, he lost weight due to diarrhea. However, after the drug was reduced to half the dose, he was able to eat more and more.”*

#### Acquisition of medical knowledge

Several participants have gained understanding of lab results for recognizing the progression of a patient’s condition. They also tried to understand the effect of the medicine by asking the question to healthcare providers.

*D: “I cannot ask the doctor if I do not understand my husband’s treatment therapy. I do not think I will be able to communicate well if I do not.”*

*G: “The doctor marked the lab results…So from then on, I try to look at the blood lab results as much as possible.”*

### Theme 2: deepening view of life and death

This theme demonstrated gradual acceptance of the patient’s current condition as participants with cancer and health problems strive to understand the patient’s experience themselves and as they become aware of death as the disease progresses. They also began forming a value system for their treatment, hoping that the treatment would continue to the extent that they would not be overwhelmed when considering the rest of their lives. In other words, their view of life and death deepened as they were confronted with the opportunity to consider how to live until death.

#### Consciousness of death

Because of the patient’s older age and the advanced stage of cancer, all participants were aware of the possibility of patient’s death. They were prepared for the fact that the disease had already progressed, even as they were undergoing palliative chemotherapy.

*F: “I was prepared for his death when I discovered his cancer had metastasized. When the doctor told me he had terminal cancer, I knew he would die soon.”*

*I: “I think I tell myself that I must think about her death. It’s the life that was given to her. So it’s a preparation to get my mind in order.”*

#### Understanding the patient’s experience

The participants who provided care for the patients tried to understand their patients’ way of life by witnessing the pain associated with cancer incidence and the treatment that patients were experiencing.

*C: “He did not need to take any more anticancer drugs because he had lost his appetite due to side effects symptoms…I want him to continue the treatment, but if he wants to quit, I have no choice but to understand his wishes.”*

#### Changing values for treatment

The experience of living with and caring for patients undergoing treatment gradually shifted their values toward treatment, from wanting patients to undergo treatment aggressively to wanting patients to undergo treatment to the extent that they are not overwhelmed and hoping that they would be able to live the rest of their lives with ease. Therefore, understanding the experiences of older spouses in depth is necessary.

*G: “I want him to continue treatment as much as possible. However, I have seen how painful the side effects and symptoms of the treatment are. Therefore, I would like the intensity of the treatment to be reduced so that he can live an easier life.”*

### Theme 3: anxious about the future

This theme referred to anxiety about the future. Participants were conflicted between hope for the effectiveness of treatment and fear about the progression of the patient’s disease. In addition, they were conflicted about cancer as a disease and their lives as a caregivers, wishing to maintain their existing lives while constantly worrying about whether their health condition was deteriorating. Above all, the participants cared for their health problems, fearing that their health problems would worsen.

#### Conflicts between treatment efficacy and Disease progression

The participants were hopeful about the effects of palliative chemotherapy for cancer but feared that their condition would worsen, and they lived with this conflict every day.

*D:“I always felt down, and …I also felt that I wanted to have hope.”*

#### Mixed feelings of desire to support recuperation and anxiety

Participants had the intention to take care of the patient. However, they also had anxiety about the difficulties of continuing palliative chemotherapy for cancer because of its side effects.

*I: “I wonder how long patients can stay in treatment. Sometimes they do not look well because they can’t eat and lose weight, and you can tell they are not doing well. So I wonder how long I can continue the treatment because I see that.”*

#### Worry about their own health

Participants themselves had chronic diseases. Therefore, they felt anxious about their health problems worsening and taking care of the patient with their health condition.

*B: “What if I have a terrible history (high blood pressure, brain aneurysm, etc…)? I do not know at my age what I would do if I had a history of high blood pressure or a cerebral aneurysm or if I should die. I always think about that.”*

### Theme 4: desire for a better rest of life

This theme was observed in all older participants, who stated that they wanted to spend their remaining time together well and that their marital bond was strengthened through the experience of caregiving. Additionally, to realize this prosperous remainder of lives, they need their children, grandchildren, and other important people to help them live. Therefore, they need connections with society including those with their families.

#### Values the remaining time

The participants who supported the patients were older adults and had a solid desire to cherish the remainder of their lives spent with patients who did not have a long life ahead and wished to stay in familiar places as long as possible. They also wanted to support the patients in their work, hobbies, and other activities even while they were undergoing treatment.

*E: “I wanted the patient to stop working when he was diagnosed with cancer. However, now, I want to let them work until they say so.”*

*F: “I want to let him do what he wants while his body can still move. We travel by car, too. That is how we like to go out together.”*

#### Energy source for living

Participants found that being involved with their children and grandchildren helped alleviate some of the hardship in caregiving. In addition, patients receiving adequate treatment and their good health were a source of energy for the participants.

*D: “My grandson is now four years old, and watching him is just the right situation to take my mind off things, and it’s good medicine for me.”*

*F: “I am so glad that the treatment was effective. After that, my husband got better, and I got better too. When his cancer recurred, I felt down, but when I heard the good results, my spirits were lifted.”*

#### Deepening affection

Through the experience of caring for the patient, the participants felt a strong desire to “do something” for the patient. In addition, they shared that the bond between the couple was strengthened by supporting the patient through his or her medical treatment.

*I:“My wife is sick, and I want to do something to help her…”.*

*J: “After all, we are a married couple…now we have to walk hand in hand, and our bond has deepened when I think about it.”*

#### Coexistence with society

As older people, the participants who care for their patients know they are supported by other family members and healthcare providers. However, they did not want to inconvenience family members, especially their children, and were searching for a way to coexist with society.

*E: “My son takes time off work to drive me to the hospital, so he helps me in that sense.”*

*I: “My sons are nearby, but I honestly do not want to bother them by having them take care of me.”*

## Discussion

This study aimed to identify the pattern in the experiences of older spousal caregivers supporting patients with cancer undergoing palliative chemotherapy. The novelty of this study is the focus on older spousal caregivers who are the primary caregivers of patients with cancer. Increasing age of the caregivers is a significant challenge due to the aging population worldwide with increasing life expectancy and medical science advances. Our findings suggest that the experiences of older spousal caregivers fell into several themes including gradually becoming accustomed to the life as a caregiver for a patient undergoing cancer treatment, a clearer view of life and death as they face the patient’s imminent death, and a desire to spend their remaining life better together as a couple despite future uncertainty. These themes are discussed in light of previous studies.

Most participants in this study described their experience of gradually adjusting to life as a part of their life rather than something special as they continued caring for these patients. For taking care of the patient, the older spouses were positively practicing caregiving in the early days of the patient’s chemotherapy, making easy-to-eat foods to cope with side effects, and checking on the patient’s physical condition. However, they gradually returned to a lifestyle similar to that before the chemotherapy sessions. Similar results were obtained in a survey of older family caregivers after patients with cancer were discharged from the hospital [28]. For caregivers of patients with cancer, the most critical concern for the quality of life has been reported to be the burden of caregiving [29]. Moreover, caregiving stress affects older caregivers more than younger ones [20]. Therefore, older spouses in this study practiced ways to reduce the burden of caregiving as they restructured their lives. To rebuild their lives, spousal caregivers need to gain support, understand the side effects of chemotherapy, and acquire knowledge and skills to cope with cancer-related symptoms and side effects through interaction with healthcare professionals [30, 31]. Moreover, the participants hid their feelings to avoid offending the patients by accommodating their wishes whenever possible to avoid stressing them. This result is supported by previous research on the characteristics of family caregivers of patients with cancer [12, 32]. In other words, through these planned activities in caregiving, older spousal caregivers were willing to make self-sacrificing efforts to live a peaceful life with their patients.

Furthermore, this section discusses the deepening view of life and death among older spousal caregivers. The participants in this study suggested that they were trying to understand their patient’s way of life by witnessing the pain associated with cancer and the treatment that the patients were experiencing. Williams [32] claimed that family caregivers felt the patient’s cancer was their own. In other words, the spousal caregivers tried empathizing with the patient’s pain. As a result, spousal caregivers shifted their values about treatment from wanting the patient to undergo chemotherapy aggressively to not being forced to undergo it. That is, since the patient was older and had advanced cancer, it can be presumed that spousal caregivers established a view of life and death by being confronted with the opportunity to consider the patient’s life to the point of death. Previous studies have reported that maintaining independence is essential to preserve the dignity of older patients undergoing chemotherapy [33] and that spouses of patients with cancer seek to live well with their patients until their deaths [34]. Therefore, the participants in this study acquired strengths through their caregiving experiences that enabled them to acquire ideas and behaviors that helped them to live a life that maintained the dignity of their patients. Views of life and death among older people can have both negative and positive aspects [35], and views of life and death influence their willingness to discuss the future [36]. Thus, for spousal caregivers supporting patients with cancer undergoing chemotherapy, the process of forming a view of life and death is essential. These considerations allow the spousal caregiver to consider with the patient’s will to receive treatment.

The participants also experienced anxiety about the uncertain future and their desire for a fulfilling remainder of their lives. The participants in this study worried about their own disease progression each time they underwent evaluation as the patient’s cancer was in an advanced stage. Furthermore, they were concerned that the progression of the disease would result in discontinuation of the current treatment. Morris [30] reported that caregivers of patients undergoing chemotherapy in an outpatient setting have fears and anxieties about the patient’s disease journey and uncertain future, and similar observations were reported in the present study. Factors related to the burden of caregiving have already been reported; these include severity of cancer symptoms [37], activities of daily living [38], and chemotherapy. This impact of caregiving on quality of life is supported by the most recent review [23]. Moreover, a significant feature of this study was that the participants, as older caregivers, expressed concern about their health issues worsening. Stolz-Baskett [39] reported that health burdens affect the psychological morbidity of older caregivers; thus, participants with chronic diseases are concerned about their ability to continue to support patients. In comparison, they also mentioned positive experiences in caregiving due to the effectiveness of treatment and the support they received from their children, grandchildren, and healthcare providers. In several previous studies, caregivers have indicated that they are satisfied with support from family and friends [32], have positive experiences with the care of healthcare providers [33], and expect to receive psychological support from health care providers for themselves as caregivers [12]. These results indicate that, similarly to the participants in this study, caregivers need to coexist with society and have familiar people including family members around them. As described above, the participants focused on the present with mixed feelings of positivity and anxiety from continuing to care for their loved ones. Moreover, as a result of the advanced stage of the patient’s cancer and the fact that both the patients and the participants were older adults, they recognized the value of their remaining time. They wished to have richer remainder of their lives. Thus, this study highlighted that the deepening of the couple’s bond through the experience of caregiving is necessary for a fulfilling remainder of life.

### Strengths and limitations of this study

This study can contribute to understanding the pattern in the experiences of older spousal caregivers supporting patients with cancer. However, there are several limitations of this study. First, the participants were experienced spousal caregivers of patients with advanced cancer undergoing palliative chemotherapy at the time of the study. Therefore, a longitudinal study would be needed to follow how the experience evolved as the patient’s subsequent medical condition and physical status deteriorated, or the caregiver’s health problems worsened. Second, the participants included caregiver for patients with advanced-stage cancer; however, these participants were able to spend some time independently at home with activities of daily living. Therefore, further studies need to be conducted with adjusted study populations, including spousal caregivers caring for patients with more advanced disease stages. Finally, the duration and length of the interviews were short. However, we are confident about the reliability of the themes, as repeated discussions of the analysis among our researchers did not affect the quality or richness of the data. Despite these possible limitations, the older spousal caregivers of patients undergoing palliative chemotherapy may find the study helpful in establishing a lifestyle and sense of values appropriate to their physical and mental state through continued caregiving. Notably, Japan has one of the highest aging population worldwide, and many patients with cancer and their spouses are older adults. Therefore, we hope that the study will contribute to a better understanding of the domain of older spousal caregivers supporting patients with advanced cancer.

### Implications for practice

The results of this study revealed that older spousal caregivers supporting patients undergoing palliative chemotherapy cared for patients while also experiencing health issues themselves. Furthermore, the participants’ commitment to the effectiveness of treatment in the face of the patient’s gradual imminent death is noteworthy. In a recent study on treatment expectations among patients with cancer and ≥ 70 years of age receiving palliative chemotherapy, 7% of patients expected to be cured, compared with 36% of the family caregivers [40]. Thus, there may be discrepancies between the perceived expectations of the patients and family caregivers [41], presuming that spousal caregivers have unmet needs. Caregivers of chemotherapy patients need education about the treatment and support in coping with it [42]. Thus, more attention is needed for awareness of the patient’s medical condition, managing the side effects of treatment, and the support system of the patient and caregiver, as perceived by the older spousal caregiver. Furthermore, healthcare professionals can help in identifying and resolving the unmet needs of patients and older spousal caregivers.

## Conclusion

Older spousal caregivers focused on the present with anxiety about their health problems worsening in the course of providing care and fear about the progression of the patient’s diseases. Above all, since both the patient and the spousal caregiver were older, they recognized the value and importance of their remaining time. Therefore, they practiced behaviors that cared for the patient’s physical condition to spend a more comfortable remainder of their lives. Furthermore, older spousal caregivers hide their emotions to make the patient comfortable. Additionally, they built their lives while strengthening their marital bond and changed their values about palliative chemotherapy their patients were receiving through the process of caregiving. This study can contribute to understanding the challenges faced and support needed by older patients with cancer and their families.

### Electronic supplementary material

Below is the link to the electronic supplementary material.

Supplementary Material 1. Additional file 1. File format: MS Word (.docx). Title of data: Interview guide. Description of data: Example of key items in the interview guide. As appropriate, ask further questions about the participant’s narrative

Supplementary Material 2. Additional file 2. File format: MS Word (.docx). Title of data: Example of theme development. Description of data: Example of theme development. It is an example of the analysis process as Theme 1 development

## Data Availability

The datasets generated and/or analyzed in this study, as well as the raw de-identified data, are available from the corresponding author upon reasonable request.

## List of abbreviations

COREQ: Consolidated Criteria for Reporting Qualitative Research
IC recorder: integrated circuit recorder

## References

[1] Kocarnik JM, Compton K, Dean FE, Fu W, Gaw BL, Harvey JD (2022). Cancer incidence, mortality, years of life lost, years lived with disability, and disability-adjusted life years for 29 cancer groups from 2010 to 2019: a systematic analysis for the global burden of Disease Study 2019. JAMA Oncol.

[2] Phillips JL, Currow DC (2010). Cancer as a chronic Disease. Collegian.

[3] Foundation for Promotion of Cancer Research. In: cancer statistics in Japan 2021. https://ganjoho.jp/public/qa_links/report/statistics/2021_en.html. Accessed 31 Aug 2022.

[4] Toledano-Toledano F, Domínguez-Guedea MT (2019). Psychosocial factors related with caregiver burden among families of children with chronic conditions. Biopsychosoc Med.

[5] Turkoglu N, Kilic D (2012). Effects of care burdens of caregivers of cancer patients on their quality of life. Asian Pac J Cancer Prev.

[6] Kim Y, Given BA (2008). Quality of life of family caregivers of cancer survivors: across the trajectory of the Illness. Cancer.

[7] Weitzner MA, Haley WE, Chen H (2000). The family caregiver of the older cancer patient. Hematol Oncol Clin North Am.

[8] Given BA, Given CW, Kozachik S (2001). Family support in advanced cancer. CA Cancer J Clin.

[9] Williams AL, Tisch AJH, Dixon J, McCorkle R (2013). Factors associated with depressive symptoms in cancer family caregivers of patients receiving chemotherapy. Support Care Cancer.

[10] Kim Y, Baker F, Spillers RL, Wellisch DK (2006). Psychological adjustment of cancer caregivers with multiple roles. Psychooncology.

[11] Üzar-Özçeti̇n YS, Dursun Sİ (2020). Quality of life, caregiver burden, and resilience among the family caregivers of cancer survivors. Eur J Oncol Nurs.

[12] Sercekus P, Besen DB, Gunusen NP, Edeer AD (2014). Experiences of family caregivers of cancer patients receiving chemotherapy. Asian Pac J Cancer Prev.

[13] Langenberg SMCH, van Herpen CML, van Opstal CCM, Wymenga ANM, van der Graaf WTA, Prins JB (2019). Caregivers’ burden and fatigue during and after patients’ treatment with concomitant chemoradiotherapy for locally advanced Head and Neck cancer: a prospective, observational pilot study. Support Care Cancer.

[14] Wagner CD, Tanmoy Das L, Bigatti SM, Storniolo AM (2011). Characterizing burden, caregiving benefits, and psychological distress of husbands of Breast cancer patients during treatment and beyond. Cancer Nurs.

[15] Kim Y, Schulz R, Carver CS (2007). Benefit-finding in the cancer caregiving experience. Psychosom Med.

[16] Pilleron S, Soto-Perez-de-Celis E, Vignat J, Ferlay J, Soerjomataram I, Bray F (2021). Estimated global cancer incidence in the oldest adults in 2018 and projections to 2050. Int J Cancer.

[17] Ketcher D, Otto AK, Vadaparampil ST, Heyman RE, Ellington L, Reblin M (2021). The psychosocial impact of spouse-caregiver chronic health conditions and personal history of cancer on well-being in patients with advanced cancer and their caregivers. J Pain Symptom Manage.

[18] Onishi H, Onose M, Okuno S, Yae S, Mizuno Y, Ito M (2005). Spouse caregivers of terminally-ill cancer patients as cancer patients: a pilot study in a palliative care unit. Palliat Support Care.

[19] Baider L, Perez T, Kaplan De-Nour A (1989). Gender and adjustment to chronic Disease. A study of couples with colon Cancer. Gen Hosp Psychiatry.

[20] Mor V, Allen S, Malin M. The psychosocial impact of cancer on older versus younger patients and their families. 1994;74 Suppl 7:2118–27. 10.1002/1097-0142(19941001)74:7+<2118::aid-cncr2820741720>3.0.co;2-n 8087779

[21] ãMinistry. of Health, Labour and Welfare. In: Summary report of comprehensive survey of living conditions. 2019. https://www.mhlw.go.jp/english/database/db-hss/dl/report_gaikyo_2019.pdf. Accessed 21 Feb 2023.

[22] ãIsac C, Lee P, Arulappan J (2021). Older adults with chronic Illness - caregiver burden in the Asian context: a systematic review. Patient Educ Couns.

[23] Donison V, Toledano N, Sigal A, McGilton KS, Alibhai SMH, Puts M (2022). Care provided by older adult caregivers to a spouse in active cancer treatment: a scoping review. Support Care Cancer.

[24] Braun V, Clarke V (2006). Using thematic analysis in psychology. Qual Res Psychol.

[25] Braun V, Clarke V (2021). Thematic analysis: a practical guide.

[26] Tong A, Sainsbury P, Craig J (2007). Consolidated criteria for reporting qualitative research (COREQ): a 32-item checklist for interviews and focus groups. Int J Qual Heal care.

[27] Braun V, Clarke V (2021). One size fits all? What counts as quality practice in (reflexive) thematic analysis?. Qual Res Psychol.

[28] Schwartz AJ, Riedel RF, LeBlanc TW, Desai D, Jenkins C, Mahoney E (2019). The experiences of older caregivers of cancer patients following hospital discharge. Support Care Cancer.

[29] Tamayo GJ, Broxson A, Munsell M, Cohen MZ (2010). Caring for the caregiver. Oncol Nurs Forum.

[30] Morris M, Marshall-Lucette S (2017). The experience of Myeloma caregivers during home-based oral chemotherapy treatment: a qualitative study. Semin Oncol Nurs.

[31] Pethybridge R, Teleni L, Chan RJ (2020). How do family-caregivers of patients with advanced cancer provide symptom self-management support? A qualitative study. Eur J Oncol Nurs.

[32] Williams AL, Bakitas M (2012). Cancer family caregivers: a new direction for interventions. J Palliat Med.

[33] Farrell C, Heaven C (2018). Understanding the impact of chemotherapy on dignity for older people and their partners. Eur J Oncol Nurs.

[34] Hisamatsu M, Shinchi H, Tsutsumi Y (2020). Experiences of spouses of patients with cancer from the notification of palliative chemotherapy discontinuation to bereavement: a qualitative study. Eur J Oncol Nurs.

[35] Hallberg IR (2004). Death and dying from old people’s point of view. A literature review. Aging Clin Exp Res.

[36] Ke LS, Huang X, Hu WY, O’Connor M, Lee S (2017). Experiences and perspectives of older people regarding advance care planning: a meta-synthesis of qualitative studies. Palliat Med.

[37] Palos GR, Mendoza TR, Liao KP, Anderson KO, Garcia-Gonzalez A, Hahn K (2011). Caregiver symptom burden: the risk of caring for an underserved patient with advanced cancer. Cancer.

[38] Rajasekaran T, Tan T, Ong WS, Koo KN, Chan L, Poon D (2016). Comprehensive Geriatric Assessment (CGA) based risk factors for increased caregiver burden among elderly Asian patients with cancer. J Geriatr Oncol.

[39] Stolz-Baskett P, Taylor C, Glaus A, Ream E (2021). Supporting older adults with chemotherapy treatment: a mixed methods exploration of cancer caregivers’ experiences and outcomes. Eur J Oncol Nurs.

[40] Ikander T, Jeppesen SS, Hansen O, Raunkiær M, Dieperink KB (2021). Patients and family caregivers report high treatment expectations during palliative chemotherapy: a longitudinal prospective study. BMC Palliat Care.

[41] Rodenbach RA, Norton SA, Wittink MN, Mohile S, Prigerson HG, Duberstein PR (2019). When chemotherapy fails: emotionally charged experiences faced by family caregivers of patients with advanced cancer. Patient Educ Couns.

[42] Marshall VK, Cairns PL (2019). Challenges of caregivers of cancer patients who are on oral oncolytic therapy. Semin Oncol Nurs.

