# Supplementary material for: Experiences of the older spousal caregivers of patients with cancer during palliative chemotherapy: a qualitative descriptive study
Source: BMC Palliat Care. 2023 Nov 23;22:188. doi: 10.1186/s12904-023-01313-2 (PMC10666444; doi:10.1186/s12904-023-01313-2)
Supplement: Supplementary file 1 — Supplementary Material 1. Additional file 1. File format: MS Word (.docx). Title of data: Interview guide. Description of data: Example of key items in the interview guide. As appropriate, ask further questions about the participant’s narrative [file 12904_2023_1313_MOESM1_ESM.docx]

**Interview guide**

**Exemplary questions**

1. What is the nature of the care you provide to patients?

2. What are your feelings, thoughts, and support needed as you care for a patient?

3. What do you do to make a living while caring for patients?

4. What is your current disease? How do you feel about caring for a patient with a disease?

Additional file 1. Example of key items in the interview guide. As appropriate, ask further questions about the participant's narrative.
