# Supplementary material for: Experiences of the older spousal caregivers of patients with cancer during palliative chemotherapy: a qualitative descriptive study
Source: BMC Palliat Care. 2023 Nov 23;22:188. doi: 10.1186/s12904-023-01313-2 (PMC10666444; doi:10.1186/s12904-023-01313-2)
Supplement: Supplementary file 2 — Supplementary Material 2. Additional file 2. File format: MS Word (.docx). Title of data: Example of theme development. Description of data: Example of theme development. It is an example of the analysis process as Theme 1 development [file 12904_2023_1313_MOESM2_ESM.docx]

**Example of theme development**

| **Example of extracts** | **Code** | **Sub-theme** | **Theme** |
| --- | --- | --- | --- |
| *C: “At the beginning, when the treatment started, I was the one who devised his diet. But he didn’t eat much because of the side effects, so we eventually reverted to our previous lifestyle.”* | No. 30:  Discontinuation of dietary innovations | Habituation of supporting recuperation | Getting used to living with the disease |
| *D: “My husband had a tough time at times with little appetite due to the side effects of the chemotherapy. So, I would write down what he ate for breakfast, lunch, and dinner, how much he ate, and things like that.”* | No. 47:  Preparing suitable meals with the beginning of treatment |  |  |
| *G: “I know he is sick, so I try not to get angry and react as gently as possible. And try not to argue with him because I think it is better not to provoke him too much.”* | No. 93:  Taking care to allow for patient selfishness |  |  |
| *H: “Even though I am not as aware as I used to be, I try to talk to her as much as possible about how I should help her here. For example, I used to go shopping with my wife, but now I sometimes go shopping alone. These responsibilities may have increased. I may have become more involved in these areas without realizing it.”* | No. 116:  Habitual care to reduce patient burden |  |  |
| *B: “Symptoms became progressively more severe in the fourth therapy session than in the first.”* | No. 15:  Recognition of symptom accumulation with repeated treatment | Recognition of side effects |  |
| *I: “When taking the maximum dose of Giotrif, he lost weight due to diarrhea. However, after the drug was reduced to half the dose, he was able to eat more and more.”* | No. 131:  Recognition of improved dietary intake due to the reduction of therapeutic medications |  |  |
| *D: “I cannot ask the doctor if I don’t understand my husband’s treatment therapy. I don’t think I will be able to communicate well if I don’t.”* | No. 56:  Learning to engage with healthcare providers | Acquisition of medical knowledge |  |
| *G: “The doctor marked the lab results. It was definitely a low white blood cell count. So from then on, I try to look at the blood lab results as much as possible.”* | No. 104:  Become familiar with the results of tests to understand medical conditions |  |  |

Additional file 2. Example of theme development. It is an example of the analysis process as Theme 1 development.
